# Factors associated with potentially serious incidental findings and with serious final diagnoses on multi-modal imaging in the UK Biobank Imaging Study: A prospective cohort study

**DOI:** 10.1371/journal.pone.0218267

**Published:** 2019-06-17

**Authors:** Lorna M. Gibson, John Nolan, Thomas J. Littlejohns, Edouard Mathieu, Steve Garratt, Nicola Doherty, Steffen Petersen, Nicholas C. W. Harvey, Jonathan Sellors, Naomi E. Allen, Joanna M. Wardlaw, Caroline A. Jackson, Cathie L. M. Sudlow

**Affiliations:** 1 Usher Institute of Population Health Sciences and Informatics, University of Edinburgh, Edinburgh, United Kingdom; 2 Clinical Trial Service Unit and Epidemiological Studies Unit, University of Oxford, Oxford, United Kingdom; 3 Nuffield Department of Primary Care Health Sciences, University of Oxford, Oxford, United Kingdom; 4 UK Biobank Co-ordinating Centre, UK Biobank, Stockport, United Kingdom; 5 William Harvey Research Institute, Queen Mary University of London, London, United Kingdom; 6 Barts Heart Centre, Barts Health NHS Trust, London, United Kingdom; 7 MRC Lifecourse Epidemiology Unit, University of Southampton, Southampton, United Kingdom; 8 UK Dementia Research Institute, University of Edinburgh, Edinburgh, United Kingdom; 9 Centre for Clinical Brain Sciences, University of Edinburgh, Edinburgh, United Kingdom; McLean Hospital, UNITED STATES

## Abstract

**Background:**

Feedback of potentially serious incidental findings (PSIFs) to imaging research participants generates clinical assessment in most cases. Understanding the factors associated with increased risks of PSIFs and of serious final diagnoses may influence individuals’ decisions to participate in imaging research and will inform the design of PSIFs protocols for future research studies. We aimed to determine whether, and to what extent, socio-demographic, lifestyle, other health-related factors and PSIFs protocol are associated with detection of both a PSIF and a final diagnosis of serious disease.

**Methods and findings:**

Our cohort consisted of all UK Biobank participants who underwent imaging up to December 2015 (n = 7334, median age 63, 51.9% women). Brain, cardiac and body magnetic resonance, and dual-energy x-ray absorptiometry images from the first 1000 participants were reviewed systematically by radiologists for PSIFs. Thereafter, radiographers flagged concerning images for radiologists’ review. We classified final diagnoses as serious or not using data from participant surveys and clinical correspondence from GPs up to six months following imaging (either participant or GP correspondence, or both, were available for 93% of participants with PSIFs). We used binomial logistic regression models to investigate associations between age, sex, ethnicity, socio-economic deprivation, private healthcare use, alcohol intake, diet, physical activity, smoking, body mass index and morbidity, with both PSIFs and serious final diagnoses. Systematic radiologist review generated 13 times more PSIFs than radiographer flagging (179/1000 [17.9%] versus 104/6334 [1.6%]; age- and sex-adjusted OR 13.3 [95% confidence interval (CI) 10.3–17.1] p<0.001) and proportionally fewer serious final diagnoses (21/179 [11.7%]; 33/104 [31.7%]). Risks of both PSIFs and of serious final diagnoses increased with age (sex-adjusted ORs [95% CI] for oldest [67–79 years] versus youngest [44–58 years] participants for PSIFs and serious final diagnoses respectively: 1.59 [1.07–2.38] and 2.79 [0.86 to 9.0] for systematic radiologist review; 1.88 [1.14–3.09] and 2.99 [1.09–8.19] for radiographer flagging). No other factor was significantly associated with either PSIFs or serious final diagnoses. Our study is the largest so far to investigate the factors associated with PSIFs and serious final diagnoses, but despite this, we still may have missed some associations due to sparsity of these outcomes within our cohort and small numbers within some exposure categories.

**Conclusion:**

Risks of PSIFs and serious final diagnosis are substantially influenced by PSIFs protocol and to a lesser extent by age. As only 1/5 PSIFs represent serious disease, evidence-based PSIFs protocols are paramount to minimise over-investigation of healthy research participants and diversion of limited health services away from patients in need.

## Introduction

Brain and body imaging is increasingly used for research, diagnostic and screening purposes and is accompanied by the risk of identifying abnormalities which are unrelated to the purposes of the imaging, so-called incidental findings (IFs) [1]. Since very few IFs turn out to represent serious disease [2], it is of limited value to feedback clearly non-serious IFs. Therefore, we focus on potentially serious IFs (PSIFs), defined as those which indicate the possibility of a condition which, if confirmed, would carry a real prospect of seriously threatening life span, or of having a substantial impact on major body functions or quality of life [2]. Feedback of PSIFs detected during research imaging generates some form of clinical assessment (e.g. general practitioner appointments and specialist referrals, or further investigations including imaging and invasive procedures) in almost all cases [2]. Information on the factors associated with increased risk of detection and feedback of a PSIF (and therefore of subsequent clinical assessment), and with increased risk of eventually receiving a serious final diagnosis may influence individuals’ decisions to consent to participate in imaging research [3–5] and inform researchers’ designs of appropriate PSIFs policies, which are required by major research funders [6, 7].

A small number of studies (N = 151 to 5800) which followed-up unselected participants with PSIFs suggest that PSIFs are associated with age, but not with sex. However none of these studies investigated the associations of PSIFs with PSIFs protocols, or any factors associated with serious final diagnoses [8–17].

The UK Biobank Imaging Study provides an opportunity to investigate potential risk factors for PSIFs and serious final diagnoses. In the UK Biobank Imaging Study, 100,000 of the original 500,000 participants are undergoing brain, cardiac and body magnetic resonance imaging (MRI), dual-energy X-ray absorptiometry (DXA) and carotid Doppler ultrasound; over 32,000 participants have been imaged as of December 2018] [18]. These imaging data are linked to detailed sociodemographic, lifestyle, physical measurement, genetic and routine healthcare data generating an extensive research resource [19].

The UK Biobank Imaging Study will inevitably generate PSIFs. To inform the development of a pragmatic PSIFs protocol that aims to minimise harm to (the largely asymptomatic) 100,000 imaged participants, UK Biobank reviewed current practice, published literature and guidance, and sought advice from professional bodies, and from ethical and legal experts [2]. The protocol is based on radiographers flagging images of potential concern to a radiologist for their review [2, 20]. This approach was evaluated against a protocol involving systematic radiologist review of all images (which is more commonly used in research studies), and found to generate less harm (i.e., less unnecessary anxiety to participants and their families) and a lower burden on the publicly-funded UK National Health Service [2]. UK Biobank is continuing to evaluate this PSIFs protocol through systematic follow-up of all participants identified with a PSIF.

We aimed to determine whether, and to what extent, socio-demographic, lifestyle, other health-related factors and PSIFs protocol are associated with detection of both a PSIF and a final diagnosis of serious disease. We achieved this using data from the first 7,334 participants imaged during the first 20 months of the UK Biobank Imaging Study (including systematic follow-up of 283 participants with PSIFs).

## Methods

We prepared this manuscript according to STROBE guidelines (S1 File) [21]. The statistical analysis code is available online [22]. UK Biobank obtained ethics approval for the imaging study, and evaluation of the PSIFs protocol (North West Research Ethics Committee reference numbers: 11/NW/0382; 16/NW/0274). We provided all participants with written information about the imaging study and the UK Biobank imaging IFs protocol [23]. All participants provided written consent to take part in the imaging study, and for UK Biobank to feed back any identified potentially serious IFs to them and their general practitioner (GP).

### UK Biobank Imaging Study

**Fig 1:**
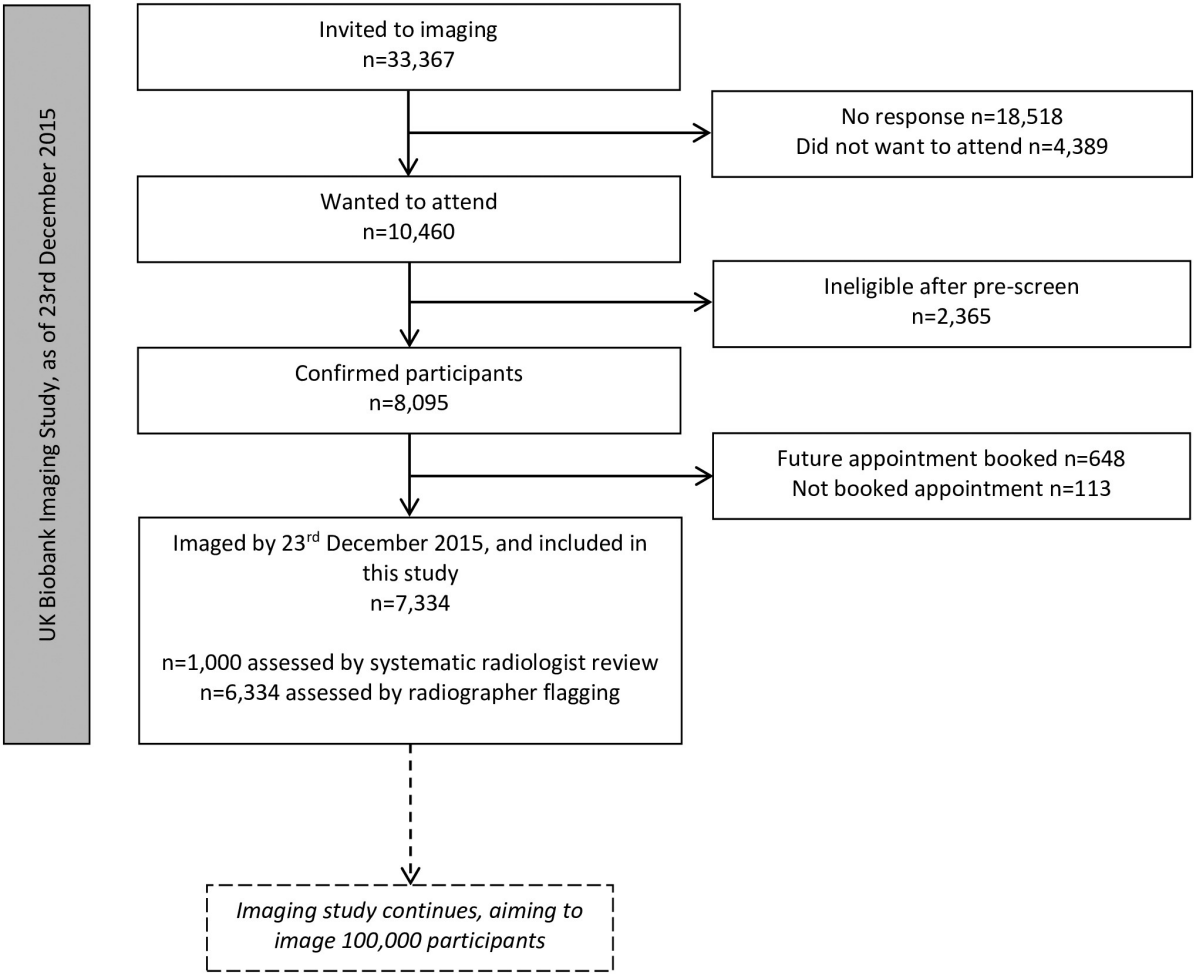
Participant flowchart.

Of 9.2 million adults aged 40–69 invited to participate in UK Biobank, 0.5 million (5.5%) participated, providing initial baseline data between 2006 and 2010 [24]. From April 2014 to December 2015, participants living within approximately 120 km of the imaging centre in Stockport were further invited to take part in the UK Biobank Imaging Study [25]. Participants were excluded if they had metal implants, penetrating metal injury, non-removable metallic items, or if they would find it difficult to complete the imaging, e.g. due to claustrophobia (Fig 1) [25].

At the imaging visit, participants underwent brain, heart and body MRI, whole-body, spine and hip DXA and carotid Doppler ultrasound [26–30]. Participants also repeated the UK Biobank baseline assessment, which involved: a touchscreen questionnaire to collect data on potentially relevant risk factors for diseases, including sociodemographic, lifestyle and medical history; an interview; and physical measurements [31].

### UK Biobank PSIFs protocol

During imaging, UK Biobank radiographers may notice PSIFs and ‘flag’ concerning images for radiologist review; radiologist-confirmed PSIFs are then fed back to participants and their GP [2]. To evaluate this PSIFs protocol, all images from the first 1000 participants were also systematically reviewed by radiologists for PSIFs [2]. Radiographers did not flag any PSIFs in addition to those detected by the radiologists within the first 1000 imaged participants [2]. Therefore, for the purposes of this present study, we classified the first 1000 imaged participants as undergoing the ‘systematic radiologist review’ PSIFs protocol, and subsequently imaged participants as undergoing the ‘radiographer flagging’ PSIFs protocol. For both protocols, to aid interpretation of images, radiologists received information on participants’ age, sex, ethnicity, alcohol intake, smoking status, blood pressure, body mass index (BMI), employment status, and self-reported medical history. The legal and ethical background to the UK Biobank ‘radiographer flagging’ protocol has been previously described [2].

Participants with PSIFs are surveyed at six weeks and six months after receiving feedback, while their GPs are surveyed six months after feedback and asked for copies of relevant clinical correspondence; these responses include data on final diagnoses [2].

Carotid Doppler ultrasound was deemed extremely unlikely to generate PSIFs under UK Biobank’s protocol [25], as asymptomatic carotid stenosis is rare and the clinical relevance is not well established, and extra-carotid abnormalities were not likely to be relevant as UK Biobank sonographers are trained in vascular Doppler US only. As such, carotid Doppler US data are not included in this study.

### Data sources and variables

#### PSIFs and serious final diagnoses

We extracted data on the number, types and body region of each participant’s PSIF(s) from radiologists’ reports. A consultant physician and an experienced clinical radiology specialty trainee independently classified final diagnoses using all available survey data and clinical correspondence; we contacted participants and GPs by telephone where these data were insufficient to classify final diagnoses [2]. We classified final diagnoses as either: serious (if they were likely to threaten life span, or have a substantial impact on quality of life or major body function); not serious (if this was not the case); or indeterminate (if there remained insufficient data to classify a final diagnosis as serious or not) [2]. A PSIF which turned out to be a known diagnosis was deemed to be very unlikely have any additional impact on the participant, and therefore we classified such findings as ‘non-serious.’ classified participants with more than one PSIF according to their most serious final diagnosis [2].

#### Participant factors

We selected variables available from UK Biobank [32] which might be associated with PSIFs or would be possible confounders. These were age, sex, ethnicity, Townsend socio-economic deprivation score (which may reduce access to healthcare, increase the risk of disease and reduce opportunities for disease detection prior to research imaging), use of private healthcare (which may be associated with reduced risk of PSIFs if it increases prior knowledge of disease), alcohol intake [33], fruit and vegetable intake [34], physical activity [35], smoking status, body mass index (BMI) [36] and morbidity. We measured the latter using the Elixhauser Index calculated using Hospital Episode statistics data from two years before the date of imaging, and defined morbidity as ≥ 1 Elixhauser Index health conditions [37–39].

### Statistical analyses

Since our previous study showed that the ‘systematic radiologist review’ protocol produced approximately ten times more PSIFs compared with the ‘radiographer flagging’ protocol [2], all analyses were stratified by PSIFs protocol to control for potential confounding. We compared characteristics between participants with and without PSIFs, and with and without serious final diagnoses, and calculated age- and sex-adjusted odds ratios (ORs) with 95% confidence intervals (CIs) using binomial logistic regression models.

We tested for normal distributions of continuous variables by visual inspection of graphed data and Kolmogorov-Smirnov goodness-of-fit tests. We attempted to normalise non-normally distributed data using log transformations, and if this failed, recoded variables into categories, aiming for similar numbers of participants in each category to optimise statistical efficiency. We used non-parametric tests to compare distributions of non-normally distributed variables between two groups. We considered data to be missing if participants did not respond, or if they responded ‘do not know’ or ‘prefer not to answer’; such participants were excluded only from the relevant analyses. We present summary statistics of the characteristics of the whole UK Biobank cohort only to inform on the likely influence of factors associated with PSIFs or serious final diagnoses which may occur as the UK Biobank imaging study continues; these cannot be compared directly to the imaged sub-cohort included in this study due to lack of independence of these two samples. The majority of variables had no, or only small proportions (< 3%) of missing data. In total, 460/7334 (6.3%) participants had missing data for at least one variable. We performed all analyses using SPSS version 22.

## Results

### Participants

By 23^rd^ December 2015, 7,334 of 33,367 invited participants (22.0%) had been imaged and were included in this study (Fig 1). Median age of the imaged participants was 63 (interquartile range 56–68) years and 3,804 (51.9%) were women (Table 1).

**Table 1:** Characteristics of the UK Biobank cohort and the imaged sub-cohort included in this study.

	Entire UK Biobank cohort (of whom 100,000 will be imaged) (N = 502,205)^1^ n (%)	Imaged UK Biobank sub-cohort included in this study (N = 7,334)^2^ n (%)
**Sociodemographics**		
Age^3^		
Median (IQR)	63 (55–68)	63 (56–68)
Sex^4^		
Female	273,224 (54.4)	3804 (51.9)
Male	228,981 (45.6)	3530 (48.1)
Ethnicity^5^		
White	472,493 (94.1)	7023 (95.8)
Minority ethnicity groups	27,012 (5.4)	225 (3.1)
TDI^5^		
Median (IQR)	-2.1 (-3.6–0.6)	-2.5 (-3.9 –-0.5)
Private healthcare^5^		
Never used	120,934 (70.1)	5377 (73.3)
Ever used	49,980 (29.0)	1850 (25.2)
**Lifestyle**		
Alcohol^5^^,^^6^		
None	19,942 (14.1)	848 (11.6)
Moderate	73,886 (52.3)	4124 (56.2)
Hazardous	34,980 (24.8)	1854 (25.3)
Harmful	9,084 (6.4)	376 (5.1)
Smoking^5^		
Never	273,400 (54.4)	4350 (59.3)
Previous	172,980 (34.4)	2575 (35.1)
Current	52,947 (10.5)	319 (4.3)
Fruit and vegetable portions/day^5^^,^^7^		
< 5	342,833 (68.3)	5028 (68.6)
≥ 5	144,064 (28.7)	2141 (29.2)
Days/week of moderate physical activity^5^^,^^8^		
0–2	169,162 (33.7)	2149 (29.3)
3–4	118,615 (23.6)	1967 (26.8)
5–7	187,251 (37.3)	2983 (40.7)
**Other factors**		
Morbidity^9^		
None	457,301 (91.1)	6422 (87.6)
≥1 condition	44,904 (8.9)	912 (12.4)
BMI^5^^,^^10^		
Underweight	2625 (0.5)	47 (0.6)
Normal	162,348 (32.3)	2733 (37.3)
Overweight	212,064 (42.2)	3061 (41.7)
Obese	122,228 (24.3)	1454 (19.8)

IQR = interquartile range, TDI = Townsend Deprivation Index (higher score indicates greater deprivation), BMI = body mass index

^1^. Data collected at recruitment visit, unless otherwise indicated.

^2^. Data collected at the imaging visit, unless otherwise indicated.

^3^. Age on 30^th^ April 2014, i.e. the start of the imaging study, for the entire cohort, and the imaged cohort.

^4^. Sex data were only available from the recruitment visit.

^5^. Data were missing for ethnicity (2,700/502,205 [0.5%], 86/7,334 [1.2%]), TDI (627/502,205 [0.1%], 0/7,334 [0.0%]), private healthcare use (1,694/172,608 [1.0%, questions on private healthcare were introduced partway through the recruitment period on 29^th^ April 2009, thus giving a smaller denominator], 107/7,334 [1.5%]), alcohol (3,357/141,149 [2.3%, questions on subtypes of alcoholic drinks were introduced partway through the recruitment period on 29^th^ August 2009, thus giving a smaller denominator], 132/7,334 [1.8%]), smoking (2,878/502,205 [0.6%], 90/7,334 [1.2%]), fruit and vegetable intake (15,308/502,205 [3.0%], 165/7,334 [2.2%]), physical activity (27,177/502,205 [5.4%], 235/7,334 [3.2%]), BMI (2,940/502,205 [0.6%], 39/7,334 [0.5%]), from the whole UK Biobank cohort versus the imaged sub-cohort respectively.

^6^. We calculated alcohol intake in units per week and categorised these using British Medical Association guidelines (women: moderate > 0 < 14, hazardous 14–35, harmful > 35; men: moderate >0 < 21, hazardous 21–50, harmful > 50) [33].

^7^. We calculated portions of fruit and vegetable intake per day, and categorised these into five or more portions per day, or not. [34]

^8^. Participants were asked ‘in a typical week, on how many days did you do 10 minutes or more of moderate physical activities like carrying light loads, cycling at normal pace (do not include walking)?’ [35].

^9^. We calculate morbidity using an Elixhauser Index score [37, 38] based on two-years of routinely collected Hospital Episode Statistics data, looking back from date of recruitment for the entire UK Biobank cohort, and the date of imaging for the imaged sub-cohort. Routinely collected health data are used to calculate payments for providers for services delivered for different conditions. The system for applying prices to healthcare services changed in 2012 [39], therefore the numbers of conditions coded in health records may not be directly comparable between the entire cohort, and the imaged cohort.

^10^. We defined BMI categories as underweight, normal, overweight and obese as BMIs of <18.5, ≥18.5 < 25.0, ≥ 25.0 < 30.0, ≥ 30.0 respectively [36].

Compared to the entire UK Biobank cohort, this imaged sub-cohort included lower proportions of women, people of minority ethnicity groups, and people with less healthy lifestyles, including those with harmful alcohol intake, current smokers, low physical activity levels, or those who were overweight or obese. Conversely, a higher proportion of the imaged sub-cohort had one or more health conditions as measured using the Elixhauser Index compared to the whole cohort (Table 1).

### PSIFs and final diagnoses

PSIFs were detected in 283/7,334 (3.9%) people: 179 of the first 1000 (17.9%) by systematic radiologist review; 104 of the subsequent 6,334 (1.6%) by radiographer flagging (OR for systematic radiologist review versus radiographer flagging: 13.3, 95% CI 10.3–17.1, p<0.001, Table 2). The majority of PSIFs were finally diagnosed as clinically non-serious (229/283, 80.9%). Serious final diagnoses occurred in 54/7,334 (0.7%) participants: 21 of the first 1000 (2.1%) undergoing the systematic radiologist review protocol and 33 of the 6,334 (0.5%) undergoing the radiographer flagging protocol (OR 4.2, 95% CI 2.4–7.4, p<0.001, Table 2). Radiographer flagging thus resulted in a higher proportion of PSIFs with serious final diagnoses than radiologist review (33/104 [31.7%] versus 21/179 [11.7%] respectively). The most common serious final diagnoses were tumours and vascular diseases (Tables 3, 4 and 5). The two doctors agreed on the initial classification of final diagnoses in 270/283 (95.4%) of cases, and readily resolved the 13 cases of disagreement through discussion.

**Table 2:** Odds ratios for potentially serious incidental findings (PSIFs) and serious final diagnoses comparing two protocols.

	Systematic radiologist review (N = 1000) n (%)^1^	Radiographer flagging (N = 6334) n (%)^1^	OR (95% CI)^2^ systematic radiologist review versus radiographer flagging	p-value^3^
**PSIFs**	179 (17.9)	104 (1.6)	13.3 (10.3–17.1)	<0.001
Brain MRI	23 (2.3)	35 (0.6)	4.3 (2.5–7.3)	<0.001
Cardiac MRI	81 (8.1)	29 (0.5)	19.7 (12.8–30.2)	<0.001
Body MRI	83 (8.3)	27 (0.4)	21.3 (13.7–33.0)	<0.001
DXA	14 (1.4)	16 (0.3)	5.8 (2.8–11.9)	<0.001
**Serious final diagnoses**	21 (2.1)	33 (0.5)	4.2 (2.4–7.4)	<0.001
Brain MRI	4 (0.4)	13 (0.2)	2.0 (0.7–6.2)	0.221
Cardiac MRI	13 (1.3)	10 (0.2)	8.5 (3.7–19.5)	<0.001
Body MRI	3 (0.3)	5 (0.1)	4.1 (1.0–17.1)	0.056
DXA	1 (0.1)	5 (0.1)	1.3 (0.2–11.0)	0.818

OR = odds ratio, CI = confidence interval, PSIFs = potentially serious incidental findings, MRI = magnetic resonance imaging, DXA = dual energy X-ray absorptiometry

^1^. Numerators are the number of participants with at least one PSIF per region. Multiple PSIFs occurred in four participants (who had two PSIFs each) under radiographer flagging, and in 33 (28 had two and five participants had three PSIFs each) under systematic radiologist review, giving a total of 325 PSIFs; therefore the sums of the body region PSIFs are greater than the 104 and 179 participants with at least one PSIF respectively. No participant had more than one serious final diagnosis.

^2^. Age- and sex-adjusted ORs for PSIFs and serious final diagnoses.

^3^. p-value from Wald test.

**Table 3:** Serious final diagnoses.

Image modality	Serious final diagnoses	Systematic radiologist review (N = 1000) n participants	Radiographer flagging (N = 6334) n participants
Brain MRI	Arachnoid cyst with hydrocephalus	1	-
	Arteriovenous malformation	-	1
	Cavernoma	-	1
	Meningioma requiring surgery	1	3
	Normal pressure hydrocephalus	-	1
	Pituitary tumour	2	4
	Pleomorphic adenoma requiring surgery	-	1
	Vestibular schwannoma	-	2
Cardiac MRI	Atrial fibrillation	1	1
	Cardiomyopathy	2	3
	Coronary heart disease	1	-
	Heart block and LV impairment	1	-
	Lung tumour	3	-
	Mesothelioma	-	1
	Myxoma	-	1
	Severe valve disease	-	2
	Thoracic aortic aneurysm	5	2
Body MRI: Abdomen	Abdominal aortic aneurysm > 5 cm	1	1
	Colonic tumour	-	1
	Gastrointestinal stromal tumour	1	-
	Pancreatic tumour	1	1
	Renal tumour	-	2
DXA	Osteoporotic crush fracture	1	5
**All modalities: serious final diagnoses**		**21**	**33**

MRI = magnetic resonance imaging, LV = left ventricular, DXA = dual-energy X-ray absorptiometry,— = zero

**Table 4:** Non-serious final diagnoses.

Image modality	Non-serious final diagnoses	Systematic radiologist review (N = 1000) n participants	Radiographer flagging (N = 6334) n participants
Brain MRI	Already known diagnosis	1	3
	Benign cyst/lesion	15	10
	Hydrocephalus (not serious)	-	2
	Suspected lesion not confirmed	3	3
Cardiac MRI	Already known cardiac diagnosis	7	5
	Already known lung diagnosis	2	1
	Already under investigation	-	1
	Cardiac diagnosis—not serious	8	8
	Lung diagnosis—not serious	28	2
	Other non-serious diagnosis	10	1
	Suspected lesion not confirmed	18	1
Body MRI: Abdomen	Abdominal aortic aneurysm < 5cm	2	1
	Already known diagnosis	4	3
	Benign lesion (e.g. cyst)	57	14
	Other non-serious diagnosis	4	-
	Suspected lesion not confirmed	13	2
Body MRI: Leg	Already known diagnosis	1	-
	Bone/soft tissue diagnosis—not serious	5	-
	Suspected lesion not confirmed	2	-
DXA	Already known diagnosis	5	5
	Non-serious diagnosis	5	3
	Suspected lesion not confirmed	2	2
**All modalities: non-serious final diagnoses**		**192**	**67**

MRI = magnetic resonance imaging, DXA = dual-energy X-ray absorptiometry,— = zero

**Table 5:** Uncertain final diagnoses.

Image modality	Uncertain final diagnoses	Systematic radiologist review (N = 1000) n participants	Radiographer flagging (N = 6334) n participants
Brain MRI	Lesion, unclear nature	-	4
Cardiac MRI	Lung consolidation, unclear nature	1	1
	Lung nodule, unclear nature	2	-
Body MRI: Abdomen	Cysts, unclear nature	-	2
DXA	Crush fracture T11, unclear relevance	1	-
	Fractures, unclear cause	-	1
**All modalities: uncertain final diagnoses**		**4**	**8**

MRI = magnetic resonance imaging, DXA = dual-energy X-ray absorptiometry,— = zero

Systematic radiologist review generated higher proportions of PSIFs on all imaged body regions (OR range 4.3–21.3, all p<0.001, Table 2) compared to radiographer flagging. Radiologists more commonly detected PSIFs on cardiac (8.1%) and body MRI (8.3%) compared to brain MRI (2.3%) or DXA (1.4%). In contrast, radiographer flagging generated similar proportions of PSIFs across body regions (range 0.3–0.6%, Table 2). Serious final diagnoses occurred most commonly on cardiac MRI assessed by systematic radiologist review (13/1000, 1.3%, Table 2).

### Factors associated with PSIFs and serious final diagnoses

**Fig 2:**
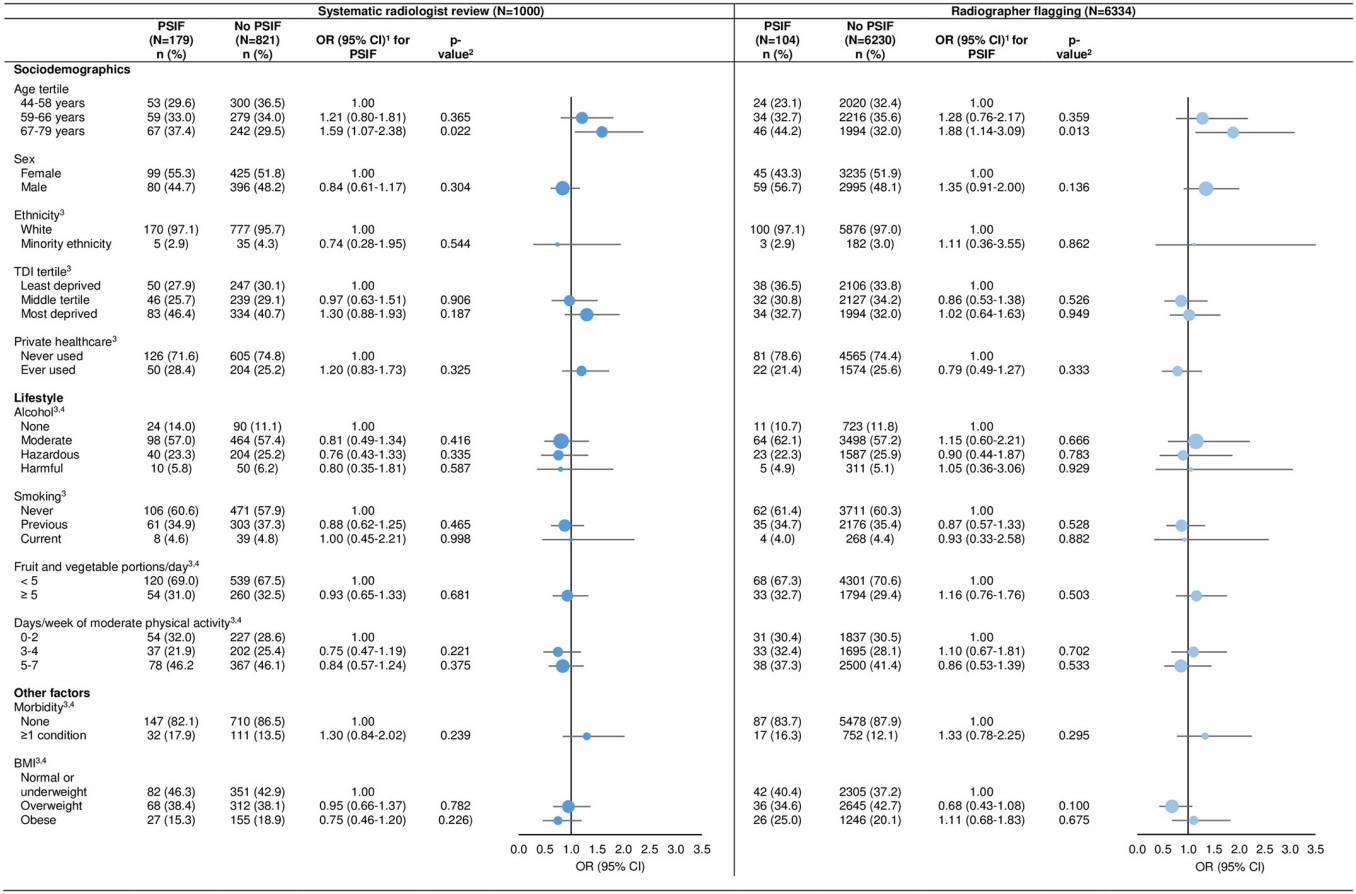
Age- and sex-adjusted odds ratios for potentially serious incidental findings (PSIFs) stratified by PSIFs protocol. PSIFs = potentially serious incidental findings, OR = odds ratio, CI = confidence interval, TDI = Townsend Deprivation Index, BMI = body mass index. Circles are weighted by the proportion of participants within a category. 1. Age- and sex-adjusted ORs, except age tertiles which are adjusted for sex only, and sex which is adjusted for age only. 2. p-value from Wald test. 3. Data were missing for ethnicity (13/1000 [1.3%], 73/6334 [1.2%]), TDI (1/1000 [0.1%], 3/6334 [<0.0%]), private healthcare use (15/1000 [1.5%], 92/6334 [1.5%]), alcohol (20/1000 [2.0%], 112/6334 [1.8%]), smoking (12/1000 [1.2%], 78/6334 [1.2%]), fruit and vegetable intake (27/1000 [2.7%], 138/6334 [2.2%]), physical activity (35/1000 [3.5%], 200/6334 [3.2%]) and BMI (5/1000 [0.5%], 34/6334 [0.5%]), for participants assessed by systematic radiologist review and by radiographer flagging respectively. 4. We calculated alcohol intake, fruit and vegetable intake, physical activity, morbidity and BMI as described in the footnotes to Table 1.

**Fig 3:**
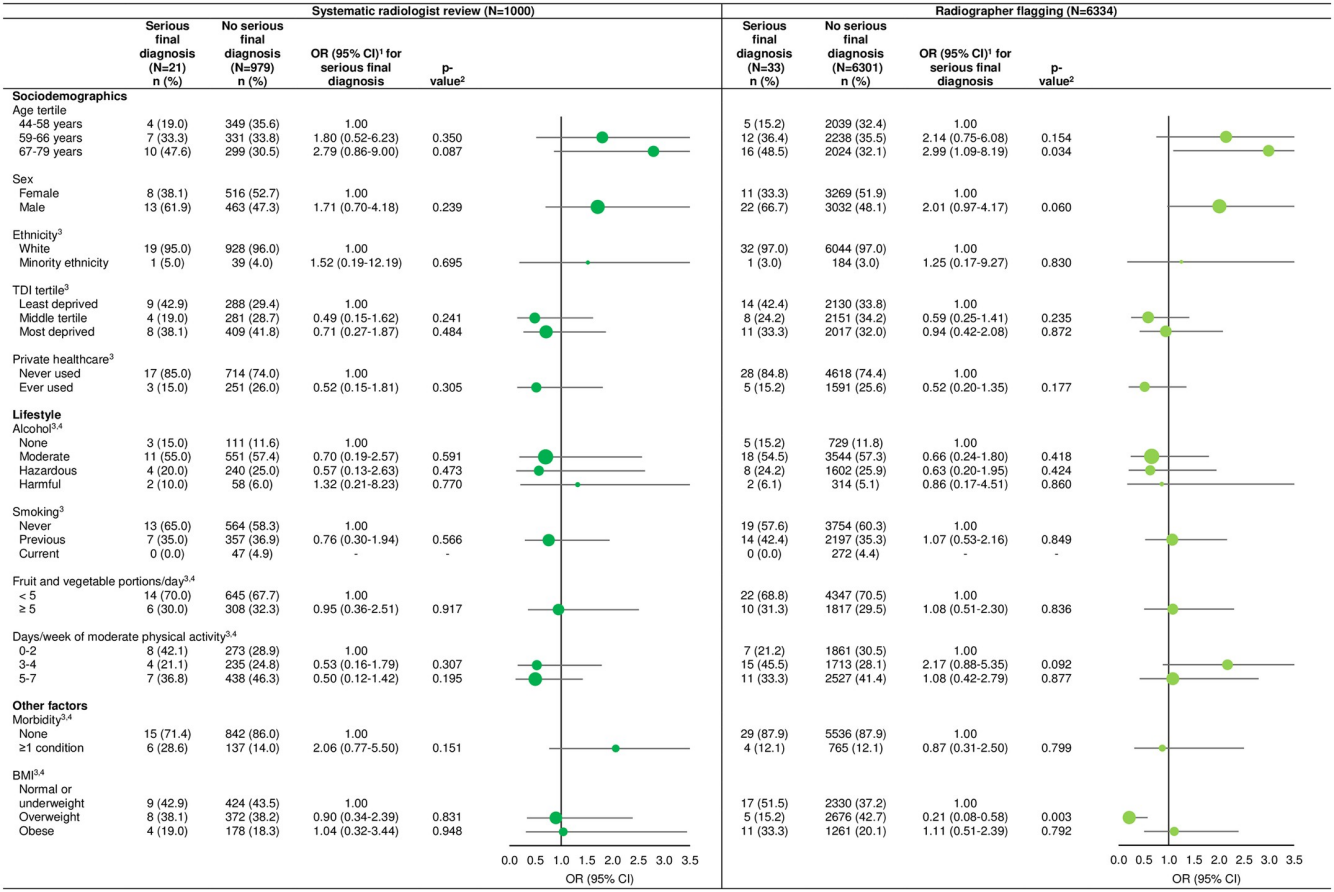
Age- and sex-adjusted odds ratios for serious final diagnoses stratified by PSIFs protocol. OR = odds ratio, CI = confidence interval, TDI = Townsend Deprivation Index, BMI = body mass index. Circles are weighted by the proportion of participants within a category. 1. Age- and sex-adjusted ORs, except age tertiles which are adjusted for sex only, and sex which is adjusted for age only. 2. p-value from Wald test. 3. Data were missing as described in Fig 2, footnote 3. 4. We calculated alcohol intake, fruit and vegetable intake, physical activity, morbidity and BMI as described in the footnotes to Table 1.

Across the relatively narrow age range of the included participants, older age was associated with an increased odds of PSIFs and of serious final diagnoses under both protocols, albeit not statistically significant for serious final diagnoses under systematic radiologist review (sex-adjusted ORs [95% CI] for oldest [67–79 years] versus youngest [44–58 years] participants for PSIFs and serious final diagnoses respectively: 1.59 [1.07–2.38] and 2.79 [0.86 to 9.0] for systematic radiologist review; 1.88 [1.14–3.09] and 2.99, 95% CI [1.09–8.19] for radiographer flagging) (Figs 2 and 3). Of the participants with PSIFs, those with serious final diagnoses were older than those with non-serious final diagnoses (median ages [range minimum-maximum] in years: 66 [50–76] versus 64 [44–76] respectively, p = 0.021).

Of participants assessed by radiographer flagging, overweight participants had reduced odds of serious final diagnoses compared to those of normal or underweight BMI (age- and sex-adjusted OR 0.21, 95% CI 0.08–0.58, p = 0.003, Fig 3), but the number of overweight participants was very small (n = 5).

No significant associations were found between PSIFs or serious final diagnoses and any other investigated factor for participants assessed by either PSIFs protocol (Figs 2 and 3).

## Discussion

Systematic radiologist review of images resulted in approximately 13 times more PSIFs, and four times more serious final diagnoses than the radiographer flagging protocol; these effect sizes are larger than those of any other risk factor assessed for association with either PSIFs or serious final diagnoses. Most (80%) PSIFs did not turn out to represent serious disease. The odds of PSIFs and of serious final diagnoses increased with age, regardless of PSIFs protocol. There were no clear associations between either PSIFs or serious final diagnoses and sex, ethnicity, socio-economic deprivation, use of private healthcare, alcohol intake, diet, physical activity, smoking status, BMI or morbidity among participants assessed using either PSIFs protocol.

Our study confirms and updates our previous findings from the first 1000 imaged UK Biobank participants [2]: compared to systematic radiologist review, radiographer flagging resulted in substantially fewer participants with potentially serious IFs and a higher proportion of these had serious final diagnoses. We also confirm the findings of the above-mentioned smaller cohort [2], that around 80% of PSIFs do not turn out to represent serious disease. Previous studies, mostly of brain MRI, found that PSIFs were associated with increased age [10, 13, 15, 17], but not clearly associated with sex [8–16]. We have further confirmed these findings in participants undergoing multimodal imaging of multiple body regions, and shown this to be independent of the IFs protocol. Previous studies did not demonstrate any associations with PSIFs and medical history of cardiac disease [10], psychotic episodes [40] or human-immunodeficiency virus [41]. Given the varying nature of PSIFs (tumours, aneurysms etc.), a common biological risk factor seems unlikely. Instead, we captured morbidity using the Elixhauser Index, which comprises 30 conditions [37, 38]. There was no convincing association between morbidity and either PSIFs or serious final diagnoses, but sparse data on both of these outcomes and exposure data on morbidity (which may be secondary to healthy volunteer bias and a relatively short period of retrospective capture within linked hospital admissions data, chosen to limit any bias that may arise from changes in healthcare record coding practices in 2012 [39]) may have attenuated any true association. Furthermore, different definitions of morbidity may well produce different results.

Large studies are needed to investigate the factors associated with PSIFs and with serious final diagnoses, as these outcomes are relatively rare, particularly under a protocol of radiographer flagging. Our study is the largest so far to investigate the factors associated with PSIFs, and the first to investigate factors associated with serious final diagnoses, in unselected, healthy participants undergoing MRI of any body region. Our sample is approximately 25% larger than the largest previous study of factors associated with PSIFs detected on brain MRI (N = 5,800) [8] and 50 times larger than the largest previous such study of multi-region MRI (N = 148) [16]. We systematically followed-up 50% more participants for data on final diagnoses compared to the largest previous study (N = 188) [8]. Despite the size of our study, we still may have missed associations with PSIFs or final diagnoses due to sparsity of these outcomes within our cohort and small numbers within some exposure categories (e.g. minority ethnicity groups). Healthy volunteer selection bias likely affects the UK Biobank cohort, as participants are less deprived than non-participants and less likely to be obese, smoke, drink alcohol daily or have self-reported medical conditions compared to the general population [24]. The imaged cohort are then further selected, with lower proportions of people having more ‘unhealthy’ lifestyles; imaged participants have survived and also remain healthy enough to travel to the imaging centres and undergo the imaging assessment. As with all epidemiological studies which use self-reported data, our data on exposures may be further limited by reporting bias; participants may have inaccurately reported alcohol intake, smoking habits, physical activity and diet. The apparently reduced odds of serious final diagnoses in overweight participants may be spurious, secondary to data sparsity of both the outcome and the exposure. The direction of an association (if any) between increased BMI and PSIFs is unclear. The associations between increased BMI and certain cancers [42] may lead to increased risk of PSIFs and serious final diagnoses; alternatively, risks may be reduced if people with increased BMI tend not to complete all MRI sequences, or imaging of all body regions.

Our classifications of ‘serious’ final diagnoses are based on clinical judgement using data collected up to six months after feedback of a PSIF. Reaching final diagnoses of some PSIFs may take longer [2]. Feedback of PSIFs may impact on non-medical domains such as emotional wellbeing, insurance and finances and work and activities, regardless of the health-related severity of the final diagnosis [2]. ‘Severity’ of a final diagnosis is therefore inherently difficult to judge, though we did show good agreement between two independent physicians’ classifications using a medical-based definition.

By deliberately focusing our study on participants with PSIFs and serious final diagnoses our results inform on factors associated with findings which are likely to generate clinical assessment, and those with serious health consequences, respectively. While our cohort is not representative of the general population, exposure-outcome associations can be generalised to other populations [24, 43, 44], to inform the design of appropriate IFs handling policies, which are required by major funders,[7] and of materials to facilitate the informed consent of potential research participants.

Compared to sociodemographic, lifestyle and health-related factors, the protocol for identifying PSIFs protocol has by far the largest influence on the generation of PSIFs and serious final diagnoses. As the majority of PSIFs do not turn out to be serious, but feedback generates clinical assessments and negative impacts on emotional wellbeing, insurance and finances and work and activities [2], our study suggests that researchers have the opportunity to greatly influence (for better or worse) the potential harms done to participants and the burden on publicly-funded health services. There remain many unanswered questions on the impacts of different methodologies to feedback research results to participants [45]; to inform future policy design, evaluations of the impacts of different protocols are paramount.

PSIFs are rare, and few are finally diagnosed as serious disease; hence large studies are needed to investigate the associated factors. This study represents the largest such cohort so far. Furthermore, since 100,000 participants will complete the UK Biobank imaging assessment over the next few years, it will in due course be possible to update these analyses with a substantially larger sample size, providing more comprehensive and statistically better powered estimates of the factors associated with PSIFs and with serious final diagnoses.

## Supporting information

S1 File STROBE checklist. (DOCX)

## Abbreviations

BMI: Body mass index
CI: Confidence interval
DXA: Dual-energy X-ray absorptiometry
GP: General practitioner
IFs: Incidental findings
MRI: magnetic resonance imaging
OR: Odds ratio
PSIFs: Potentially serious incidental findings
