# Supplementary material for: Factors associated with potentially serious incidental findings and with serious final diagnoses on multi-modal imaging in the UK Biobank Imaging Study: A prospective cohort study
Source: PLoS One. 2019 Jun 17;14(6):e0218267. doi: 10.1371/journal.pone.0218267 (PMC6576786; doi:10.1371/journal.pone.0218267)
Supplement: S1 File — (DOCX) [file pone.0218267.s001.docx]

**STROBE Statement—checklist of items that should be included in reports of observational studies**

|  | | Item No | | Recommendation | Relevant section(s) of manuscript |
| --- | --- | --- | --- | --- | --- |
| **Title and abstract** | | 1 | | (*a*) Indicate the study’s design with a commonly used term in the title or the abstract | Title page and abstract |
|  |  |  |  | (*b*) Provide in the abstract an informative and balanced summary of what was done and what was found | Abstract |
| Introduction | | | | |  |
| Background/rationale | | 2 | | Explain the scientific background and rationale for the investigation being reported | Introduction |
| Objectives | | 3 | | State specific objectives, including any pre-specified hypotheses | Introduction |
| Methods | | | | |  |
| Study design | | 4 | | Present key elements of study design early in the paper | Methods |
| Setting | | 5 | | Describe the setting, locations, and relevant dates, including periods of recruitment, exposure, follow-up, and data collection | Methods subsections: UK Biobank Imaging Study, UK Biobank PSIFs protocol, Data sources and variables |
| Participants | | 6 | | (*a*) *Cohort study*—Give the eligibility criteria, and the sources and methods of selection of participants. Describe methods of follow-up  *Case-control study*—Give the eligibility criteria, and the sources and methods of case ascertainment and control selection. Give the rationale for the choice of cases and controls  *Cross-sectional study*—Give the eligibility criteria, and the sources and methods of selection of participants | Methods subsections: UK Biobank Imaging Study, UK Biobank PSIFs protocol, |
|  |  |  |  | (*b*) *Cohort study*—For matched studies, give matching criteria and number of exposed and unexposed  *Case-control study*—For matched studies, give matching criteria and the number of controls per case | NA |
| Variables | | 7 | | Clearly define all outcomes, exposures, predictors, potential confounders, and effect modifiers. Give diagnostic criteria, if applicable | Methods subsections: Data sources and variables. Further information is available in the footnotes to Figure 2 and Figure 3, and within statistical analyses code files |
| Data sources/ measurement | | 8* | | For each variable of interest, give sources of data and details of methods of assessment (measurement). Describe comparability of assessment methods if there is more than one group | Methods subsections: Data sources and variables. Further information is available in the legends of Figure 2 and Figure 3, and within statistical analyses code files |
| Bias | | 9 | | Describe any efforts to address potential sources of bias | Discussion, and legends of Figure 2 and Figure 3 |
| Study size | | 10 | | Explain how the study size was arrived at | Figure 1 |
| Quantitative variables | | 11 | | Explain how quantitative variables were handled in the analyses. If applicable, describe which groupings were chosen and why | Methods subsection: Statistical analyses |
| Statistical methods | | 12 | | (*a*) Describe all statistical methods, including those used to control for confounding | Methods subsection: Statistical analyses |
|  |  |  |  | (*b*) Describe any methods used to examine subgroups and interactions | Methods subsection: Statistical analyses |
|  |  |  |  | (*c*) Explain how missing data were addressed | Methods subsection: Statistical analyses |
|  |  |  |  | (*d*) *Cohort study*—If applicable, explain how loss to follow-up was addressed  *Case-control study*—If applicable, explain how matching of cases and controls was addressed  *Cross-sectional study*—If applicable, describe analytical methods taking account of sampling strategy | Methods subsection: UK Biobank PSIFs protocol |
|  |  |  |  | (*e*) Describe any sensitivity analyses | NA |
| Results | | | | |  |
| Participants | 13* | | (a) Report numbers of individuals at each stage of study—eg numbers potentially eligible, examined for eligibility, confirmed eligible, included in the study, completing follow-up, and analysed | | Figure 1 |
|  |  |  | (b) Give reasons for non-participation at each stage | | Figure 1 |
|  |  |  | (c) Consider use of a flow diagram | | Figure 1 |
| Descriptive data | 14* | | (a) Give characteristics of study participants (eg demographic, clinical, social) and information on exposures and potential confounders | | Results subsection: Participants, PSIFs and final diagnoses, Figure 2 and Figure 3 |
|  |  |  | (b) Indicate number of participants with missing data for each variable of interest | | Legends to Figure 2 and Figure 3 |
|  |  |  | (c) *Cohort study*—Summarise follow-up time (eg, average and total amount) | | Methods subsection: UK Biobank PSIFs protocol |
| Outcome data | 15* | | *Cohort study*—Report numbers of outcome events or summary measures over time | | Results subsection: PSIFs and final diagnoses |
|  |  |  | *Case-control study—*Report numbers in each exposure category, or summary measures of exposure | |  |
|  |  |  | *Cross-sectional study—*Report numbers of outcome events or summary measures | |  |
| Main results | 16 | | (*a*) Give unadjusted estimates and, if applicable, confounder-adjusted estimates and their precision (eg, 95% confidence interval). Make clear which confounders were adjusted for and why they were included | | Methods subsection: Statistical analyses. Figure 2 and Figure 3. Results subsection: Associations with PSIFs and serious final diagnoses |
|  |  |  | (*b*) Report category boundaries when continuous variables were categorized | | Figure 2 and Figure 3 |
|  |  |  | (*c*) If relevant, consider translating estimates of relative risk into absolute risk for a meaningful time period | | NA |
| Other analyses | 17 | | Report other analyses done—eg analyses of subgroups and interactions, and sensitivity analyses | | Results subsections: Participants, PSIFs and final diagnoses |
| Discussion | | | | |  |
| Key results | 18 | | Summarise key results with reference to study objectives | | Discussion |
| Limitations | 19 | | Discuss limitations of the study, taking into account sources of potential bias or imprecision. Discuss both direction and magnitude of any potential bias | | Discussion |
| Interpretation | 20 | | Give a cautious overall interpretation of results considering objectives, limitations, multiplicity of analyses, results from similar studies, and other relevant evidence | | Discussion |
| Generalisability | 21 | | Discuss the generalisability (external validity) of the study results | | Discussion |
| Other information | | | | |  |
| Funding | 22 | | Give the source of funding and the role of the funders for the present study and, if applicable, for the original study on which the present article is based | | Grant information |

*Give information separately for cases and controls in case-control studies and, if applicable, for exposed and unexposed groups in cohort and cross-sectional studies.

**Note:** An Explanation and Elaboration article discusses each checklist item and gives methodological background and published examples of transparent reporting. The STROBE checklist is best used in conjunction with this article (freely available on the Web sites of PLoS Medicine at http://www.plosmedicine.org/, Annals of Internal Medicine at http://www.annals.org/, and Epidemiology at http://www.epidem.com/). Information on the STROBE Initiative is available at www.strobe-statement.org.
